# Acknowledgement to Reviewers of *Viruses* in 2017

**DOI:** 10.3390/v10010032

**Published:** 2018-01-11

**Authors:** Viruses Editorial Office

**Affiliations:** MDPI AG, St. Alban-Anlage 66, 4052 Basel, Switzerland

Peer review is an essential part in the publication process, ensuring that *Viruses* maintains high quality standards for its published papers. In 2017, a total of 395 papers were published in the journal. Thanks to the cooperation of our reviewers, the median time to first decision was 21 days and the median time to publication was 44 days. The editors would like to express their sincere gratitude to the following reviewers for their time and dedication in 2017:
Abbas, WasimMahony, Timothy JohnAboulata, A.E.Maier, HelenaAccardi, LuisaMainou, BernardoAccardi, RositaMajumder, MousumiAdams, Sandra D.Małgorzata, ŁobockaAdriaenssens, Evelien M.Manicassamy, BalajiAghemo, Alessio MicheleMarcello, AlessandroAhi, YadvinderMarchant, DavidAit-Ali, TaharMarsh, MarkAitor, Nogalez-GonzalezMartin, M.Almazan, FernandoMartín, MargaritaAlmendral, JoseMartín-Acebes, Miguel A.Altan-Bonnet, NihanMartínez Martínez, JoaquínAlvisi, GualtieroMartínez, Miguel A.Amorim, Maria JoãoMarvin, ShaunaAnastasia, VlasovaMason, William S.Androphy, ElliotMasucci, MariaAngstmann, ChristopherMasuda, HisakoAquaro, StefanoMatsuzaki, ShigenobuArbuckle, JesseMatsuzaki, YokoArias, ArmandoMayer, JensArif, MohammadMcBride, AlisonArnold, MichelleMcCollum, AndreaAscencio-Ibanez, TrinoMcCormick, CraigAssunção-Miranda, IranaiaMcInerney, GeraldAsurmendi, SebastianMcInnes, ColinAswad, AmrMcLaughlin-Drubin, MargaretAvrani, SaritMcLellan, Jason S.Bailey, Adam LeeMcVey, David ScottBakonyi, TamasMedcalf, SharonBakre, Abhijeet A.Medin, CareyBaldridge, Megan T.Medveczky, PeterBalkema-Buschmann, AnneMei, Ya-FangBamford, ConnorMei, YangBańbura, Marcin W.Melcher, UlrichBanks, LawrenceMellits, KenBaptista, Pedro VianaMenéndez-Arias, LuisBarbu, MagdaMenon, VipinBarik, SailenMentis, AndreasBarton, DavidMercer, AndrewBashkin, James K.Mergia, AyalewBates, John T.Meseda, ClementBeard, Philippa M.Mespledes, ThibaultBeasley, DavidMessling, Veronika vonBeckett, Stephen J.Meurs, ElianeBeckham, J. DavidMillard, AndrewBeddows, SimonMlera, LuwanikaBeemon, KarenMochizuki, TomofumiBehboudi, ShahriarModhiran, NaphakBehjatnia, AkbarMoelling, KarinBelaganahalli, ManjunathaMojica, KristinaBellec, LaureMontassier, Helio J.Benfield, CamillaMoore, Matthew D.Benko, MáriaMoratelli, RicardoBergman, CaseyMoreau, HervéBerkhout, BenMorgan, GregoryBerndsen, Christopher E.Morgan, IainBertho, NicolasMoriones, EnriqueBertke, AndreaMorozov, Igor A.Best, Sonja M.Morrison, Lynda A.Bevins, CharlesMorrison, Thomas E.Bidgood, SusannaMorrison, Trudy G.Biegalke, BonitaMoser, Lindsey A.Bierle, CraigMoss, William JohnBigot, YvesMossel, EricBilger, AndreaMotwani, TinaBiswas-Fiss, Esther E.Mougel, MaryleneBlanc, GuillaumeMouland, AndrewBlock, TimothyMoutelikova, RomanaBlome, SandraMucker, Eric M.Bocharov, GennadyMühlebach, Michael D.Bodily, JasonMukhopadhyay, TuliBoehme, KarlMünz, ChristianBolling, BethanyMur, LinaBopegamage, ShubhadaMurakami, SeishiBorucki, MonicaMurata, TakayukiBose, DebojitMurovska, ModraBose, SayantanMurphy, EainBossis, IoannisMustafa, FarahBouchard, Michael J.Mutsafi, YaelBourgeois-Daigneault, Marie-ClaudeMyoung, JinjongBowen, RichardNagasaka, KazunoriBrandt, Curtis R.Nagasaki, KeizoBranza-Nichita, NoricaNardacci, RobertaBrassard, JulieNarute, PurushottamBratbak, GunnarNemerow, GlenBrault, AaronNetherton, ChrisBrault, VeroniqueNeumann, Donna M.Bravo, Ignacio G.Ng, Kenneth Kai-SingBrennan, GregNichols, DanielBriant, LaurenceNing, S.Brien, James D.Nisole, SebastienBrinton, MargoNitsche, AndreasBristol, Molly L.Noguera-Julian, MarcBroadbent, AndrewNoris, EmanuelaBrockmeier, Susan L.Novick, Richard P.Brooke, ChristopherNúñez Garrote, José IgnacioBrugnera, EnricoNuttall, Patricia A.Brulois, KevinO’Shea, HelenBrum, JenniferObbard, Darren J.Brussow, HaraldOgliastro, MylèneBukrinsky, MichaelOkamoto, ToruBullitt, EstherOkeke, Malachy IfeanyiBun NG, TziOkino, Cintia HiromiBurkard, ChristineOLESEN, NielsBurri, Dominique JulienOlson, VictoriaBusca, AureliaOno, AkiraCaffrey, MichaelOshitani, HitoshiCalisher, CharlesOsolodkin, DmitryCalvo-Pinilla, EvaOthumpangat, SreekumarCampbell, CoreyØynebråten, IngerCanini, LaetitiaOzbun, Michelle A.Cao, DianjunPachulska-Wieczorek, KatarzynaCao, Sheng-BoPadmanabhan, R. PadCao, ZhiweiPaeshuyse, JanCardeti, GiusyPaillart, Jean-ChristopheCarrasco, AdrianoPanabieres, FranckCaruso, SteveParadkar, PrasadCasjens, SherwoodParanhos-Baccalà, GlauciaCasotti, RaffaellaParcells, MarkCastillo, DanielParent, KristinCen, OsmanParet, Mathews L.Cenci, GiovanniParish, ColinČerný, JiříParish, JoannaChackerian, BryceParisi, Saverio G.Chaix, CaroleParker, AlanChajȩcka-Wierzchowska, WioletaParker, ScottChalkia, DimitraParkhouse, MichaelChang, Hui-WenParolin, CristinaChang, JunPaskaleva, ElenaChang, KCPassaes, Caroline Pereira BittencourtChapman, Nora M.Passarelli, A. LorenaChatel-Chaix, LaurentPatil, BasavaprabhuChen, Mei-RuPatterson, StevenChen, QiPawlikowska, MałgorzataChen, Shun-HuaPearson, AngelaCheng, Xiao-WenPelletier, JerryChiou, Shyan-SongPellett, PhilipChoi, Kang-SeukPenades, Jose R.Christensen, ShawnPeng, ChenChung, AmyPeng, XuChung, Brian K.Pérez Esteban, PatriciaChung, DonghoonPerilla, Juan RobertoChung, SanghyukPetit, Marie-AgnèsClark, Duncan A.Petrini, StefanoClement, SophiePfeffer, MartinCoen, Donald M.Piantino Ferreira, Antonio JoséColangelo-Lillis, JessePickup, DavidComas Garcia, MauricioPijlman, Gorben P.Comas-Garcia, AndreuPintel, DavidCooper, ChristopherPittau, MarcoCorleis, BjörnPlachter, BodoCortez, ValeriePöhlmann, StefanCosset, François-LoïcPolakowski, NicholasCôté, HélènePolinski, MarkCoulibaly, FasseliPollicino, TeresaCreamer, RebeccaPolonis, VictoriaCrespo, Mariano SánchezPolston, Jane E.Cristina, MoraruPope, WelkinCrocchiolo, RobertoPoreba, ElzbietaCrump, ColinPornillos, OwenCuestas, María LujánPossee, RobertCummins, Nathan W.Postigo, AntonioCurcio, JoanPrangishvili, DavidCysique, Lucette A.Prasad, Vidya MangalaCzosnek, HenrykPrasad, Vinayaka R.Damia, GiovannaPratelli, AnnamariaDarós, José-AntonioPrevelige, PeterDash, Paban KumarProtzer, UlrikeDastjerdi, AkbarPrusty, BrupeshDatta Chaudhuri, AmritaPyeon, DohunDavidson, Alan R.Qiu, Hua-JiDavis, April D.Qiu, JianmingDavis, David A.Qiu, XiangguoDe Andrés Cara, Damián F.Querol Audí, JordiDe Chaisemartin, LucQuiñones-Mateu, Miguel E.De La Torre, JuanRadke, Jay R.De Los Santos, TeresaRakasz, EvaDe Marco, FedericoRakotondrafara, AurelieDe Miranda, JoachimRam, Angia Sriram PradeepDe Rossi, AnitaRastelli, EugenioDe Wilde, A.H.Rausch, Jason W.Dearborn, Altaira D.Ravishankar, ChintuDebyser, ZegerRavoet, JorgenDeFilippis, Victor R.Ray, Jessica LouiseDel Re, BrunellaRay, RatnaDelgado, RafaelRead, DavidDellas, NikkiReading, Patrick C.Delpeyroux, FrancisRees, CatherineDeLuca, Neal A.Regoes, RolandDeng, XufangReguera, JuanDesdevises, YvesReid, St PatrickDevadas, KrishnakumarReid, StevenDevine, Scott E.Rein, AlanDiamond, MichaelResa-Infante, PatriciaDíaz-Sanchis, MireiaRevill, PeterDiefenbach, Russell J.Reyes Muñoz, AlejandroDiel, DiegoReynolds, JessicaDikeakos, JimmyRibeiro, BergmannDiMaio, DanielRibeiro, DanielaDing, LinRiblett, AmberDinman, JonathanRicciardi, RobertDixit, UpdeshRichert-Pöggeler, KatjaDobrovolny, Hana M.Risatti, GuillermoDokland, TerjeRivera-Bustamante, RafaelDollard, SheilaRoach, DwayneDomanska-Blicharz, KatarzynaRoan, NadiaDombrovsky, AvivRoberts, John M.K.Doorbar, JohnRoberts, SallyDorin, JuliaRobertson, ESDorner, MarcusRobinson, Christopher M.Dorrington, RosemaryRobinson, Mark W.Dowd, Kimberly A.Roden, RichardDreux, MarleneRodriguez-Morales, A.J.Drigo, MicheleRoeb, ElkeDrillien, RobertRoelke, Daniel L.Drulis-Kawa, ZuzannaRoesl, FrankDu, DongliangRoman, AnnDuensing, StefanRomanelli, Maria GraziaDuerkop, Breck A.Romeo, GiovannaDufour, NicolasRomerio, FabioD'Ugo, EmilioRomero-Brey, InésDunigan, DavidRong, L.Dunne, MatthewRos, CarlosDustin, LynnRosenberg, EugeneDutra Albarnaz, JonasRosenthal, KenDvorak, CherylRösl, FrankDyall-Smith, MikeRossman, JeremyEggink, DirkRota, Paul A.Elliman, JenniferRothenburg, StefanElliott, GillRoux, SimonElofsson, MikaelRowan, AileenEngelman, Alan N.Rubio, LuisEnomoto, MasaruRudolph, HenrietteErill, IvanRuiz, TeresaEsclatine, AudreyRümenapf, Hans TillmannEspert, LucileRusnati, MarcoEssani, KarimRussell, RodneyEvans, DavidRůžek, DanielEwan, PlantRyan, Lisa K.Ewing, AdamRyu, Wang-ShickExcoffon, Katherine J.D.A.Sachse, MartinEyre, NicholasSadigh, YasharFaaberg, Kay S.Sagan, SelenaFalk, BryceSaijo, MasayukiFang, MinSáiz, Juan-CarlosFaulkner, GeoffreySakowicz, RomanFelmlee, Daniel J.Salem, Nida' MFerguson, BrianSalvatore, FrancescoFernandes, PatriciaSalvatore, MirellaFernández, LucíaSambri, VittorioFerreira, FernandoSan Martín, CarmenFinberg, Robert W.Sandaa, Ruth-AnneFinke, StefanSandmeyer, SuzanneFiorito, FilomenaSanfaçon, HélèneFirth, Andrew E.Santiago, Mario L.Fischer-Smith, TracySantiago-Rodríguez, Tasha M.Fisher, ChrisSapp, MartinFisher, Derek J.Sardo, LucaFlamand, LouisSatheshkumar, Panayampalli SubbianFletcher, Nicola F.Sattar, SampurnaFlores, RicardoScagnolari, CarolinaFlorin, LuiseSchatz, DaniellaFoley, SophieScheel, TroelsFonseca Junior, Antônio AugustoSchelhaas, MarioFonseca, FilomenaSchellhorn, Herb E.Fontana, JuanSchildgen, OliverFranco, RodrigoSchindler, MichaelFreiberg, Alexander N.Schmelcher, MathiasFrese, MichaelSchnettler, EstherFrick, DavidSchriml, Lynn M.Fuchs, MarcSchroeder, Declan C.Fuentes, AlejandroSchubert, UlrichFuruse, YukiSchuetz, AlexandraGaglia, Marta MariaSchultz-Cherry, StaceyGaia, MorganSchurko, AndrewGallagher, Thomas M.Schuurman, Henk-JanGallardo, CarminaSchwarz, Kathleen B.Gallay, PhilippeScott, KatherineGallicchio, EmilioSeaman, MichaelGamarnik, Andrea V.Sekine, KenTaroGanesan, Latha P.Sellers, Holly S.Ganges, LlilianneSeo, Keun-SeokGarboczi, DavidSerra-Moreno, RuthGarcia-Ruiz, HernanServín-Garcidueñas, Luis EduardoGareth, BradySeto, DonaldGARIGLIO, MarisaSeuberlich, TorstenGarver, JenniferShain, Daniel H.Garzino-Demo, AlfredoShapiro, JamesGerner, WilhelmSharp, Natasha J.Geslin, ClaireShembade, NoulaGevertz, JanaShemer-Avni, YonatGimenez-Lirola, LuisShen, WeiranGoff, PeterShepherd, SamanthaGoldstone, David CharlesSherer, NathanGompels, UrsulaShi, Pei YongGonzález, JosefaShishler, JoannaGonzalez, Maria EugeniaShresta, SujanGoodier, Martin R.Shrestha, SadeepGorini Da Veiga, AnaSichero, LauraGoupil, Brad A.Silva, Laurie A.Gouttenoire, JeromeSingh, KamalendraGoyal, AshishSingh, Sunit KumarGraesser, FriedrichSinha, SangitaGraham, SheilaSlagle, BettyGrandvaux, NathalieSmagghe, GuyGrant, MichaelSmartt, Chelsea T.Graw, FrederikSmee, DonaldGrayfer, LeonSmiley, JamesGrayson, Jason M.Smith, Jason G.Grdzelishvili, ValerySmith, JessicaGreive, SandraSoderlund-Venermo, MariaGriffiths, PaulSohn, SeonghyangGrimsley, NigelSokoloski, KevinGrose, Julianne H.Solorzano, AliciaGroves, IanSominskaya, IrinaGubler, Duane J.Song, JiuzhouGuo, HaitaoSong, ZhenhuiGuo, Ju-TaoSoriano, VicenteHafenstein, SusanSpearman, PaulHammerl, Jens AndreSpencer, Juliet V.Han, MingyuanSpindler, Katherine R.Harder, TimmSrinivasan, RajagopalbabuHardies, Stephen C.Stamminger, ThomasHarmon, Karen M.Stanley, MargaretHarrach, BalázsStanway, GlynHarrison, RobertStavrou, SpyridonHartshorn, KevanStech, JürgenHartung, John StephenStedman, KennethHassan, Sherif T.S.Steenbergen, RenskeHattaf, KhalidSteinbach, FalkoHauxwell, CarolineStephens, EdwardHawley, Robert J.Steven, AlasdairHe, QigaiStevens, Scott W.Hefferon, Kathleen LauraStewart, Lucy R.Heinemann, IlkaStiasny, KarinHenderson, AndrewStorici, FrancescaHerbelin, PascalineStorrie, BrianHernández, CarmenStrand, Michael R.Hernandez-Vargas, EstebanStrandin, TomasHersperger, Adam R.Strang, Blair L.Hilgenfeld, RolfStrassmann, JoanHioe, Catarina E.Striker, RobHirai, ItaruStubenrauch, FrankHirsch, Alec J.Su, YvonneHizi, AmnonSuárez, Cinta PrietoHo, Eric S.Sudarshana, MysoreHobson-Peters, JodySummerfield, ArturHoeben, RobSun, Der-ShanHoffmann, BerndSun, XiaomingHoffmann, DonataSurapathrudu, KanakalaHohn, ThomasSusi, PetriHokynar, Kati ChristelSutter, GerdHolbrook, MichaelSutton, TroyHolmes, JacintaSvenningsen, Sine LoHong, NiSymer, David E.Hoppe-Seyler, FelixSzpara, MoriahHoppe-Seyler, KarinTabernero, DavidHorner, StacyTai, Andrew W.Horton, NancyTajima, MotoshiHosie, MargaretTakeshi, MikiHoward-Varona, CristinaTang, QiyiHu, JiafenTao, JaneHu, JianmingTattersall, PeterHuang, Jason C.Tavis, JohnHuang, Yan-JangTaylor, Harry EugeneHuckriede, AnkeTaylor, MatthewHudson, AmyTazawa, HiroshiHughes, StephenTe Velthuis, AartjanHulgan, ToddTedbury, PhilipHummel, MaryTempesta, MariaHusain, MatloobTeng, MichaelHutchinson, EdwardTerrier, OlivierHyde, JenniferTeschke, CarolynIcenogle, JosephTestoni, BarbaraIkegami, TetsuroTheilmann, DavidImperiale, MichaelThomas, JulieInaba, HiroshiThomas, Stephen J.Iorio, Ronald M.Thomasini, Ronaldo L.Isel, CatherineThomason, LynnJ. Grand, RogerThomsen, Allan RandrupJakab, FerencThormann, KaiJames, WilliamThorne, ClaireJan, Fuh-JyhThoulouze, Marie IsabelleJancovich, James K.Tietjen, IanJangra, RohitTijssen, PeterJanssen, DirkTimani, KhalidJiang, SizunTincati, CamillaJohnson, JohnTischer, KarstenJohnson, Reed F.Tomaru, YujiJonassen, ChristineTong, ShupingJones, ClintonToniolo, Antonio Q.Jones, JeremyTorres-Barceló, ClaraJones, Kathryn M.Tota, Joseph E.Jones, MelissaTőzsér, JózsefJourdan Da Silva, NathalieTraktman, PaulaJung, KwonilTrilling, MirkoJung, Tae SungTrobaugh, DerekJurak, IgorTrus, Benes LouisKaddour, HusseinTsai, Chi-WeiKainov, DenisTso, For YueKaiser, William J.Tsukiyama-Kohara, KyokoKamitani, WataruTurner, DannKanda, TatsuoUeki, ShokoKarlsson, ErikUlaeto, DavidKarst, StephanieUlbert, SebastianKashanchi, FatahUmber, MarieKataria, Jag MohanUpadhyay, AnupKaufer, BenediktUpton, ChrisKauffman, Anne Kathryn MarieUrbino, CicaKazuo, NishigakiUstav, MartKe, RuianUsui, T.Kehn-Hall, KyleneVan Den Wollenberg, Diana J. M.Keil, Günther M.Van Der Kuyl, AntoinetteKemp, Michael G.Van Doorslaer, KoenraadKennedy, PatrickVan Drunen Littel-van den Hurk, SylviaKennedy, Richard G.Van Etten, JamesKhatri, MaheshVan Geelen, AlbertKim, AlexanderVan Gorp, E.C.M.Kim, MeehyeinVan Reeth, KristienKim, Shin-HeeVan Zyl, Leonardo JoaquimKindrachuk, JasonVanet, AnneKlose, ThomasVanham, GuidoKlupp, BarbaraVaqué, DolorsKnecht, HansVardi, NoamKnezevic, PetarVarsani, ArvindKnierim, DennisVentoso, IvánKochneva, GalinaVenuti, AldoKodama, HiroakiVerjans, Georges M.G.M.Koh, ChristopherViejo-Borbolla, AbelKong, WeiliVillarreal, LuisKónya, JózsefVinjé, JanKoonin, Eugene V.Viviana, CastillaKoshiba, TakumiVoigt, SebastianKousoulas, Konstantin G.Von Bargen, SusanneKoyuncu, OrkideVon Brunn, AlbrechtKramvis, AnnaWada, RyuichiKreher, FelixWalkden-Brown, Stephen W.Kremer, E.Wallace, NicholasKrenz, BjornWan, HenryKrijnse-Locker, JacomineWan, YonghongKuang, YangWang, ChunlingKugelman, JeffWang, DanKuhn, AndreasWang, DavidKuhn, RichardWang, Fun-InKukimoto, IwaoWang, JenniferKulakov, LeonidWang, NianshuangKumar, AmitWang, QiuhongKumar, AshokWang, RanranKumar, BinodWang, XifengKumar, MukeshWang, XiuqingKuprash, DmitriyWang, YingKuroda, Shun’ichiWard, Brian M.Kutluay, Sebla B.Ward, KateKvansakul, MarcWatson, AndrewKyei, George B.Weger, StefanL’Homme, YvanWegrzyn, GrzegorzLabonté, JessicaWeibel, Sara GianellaLager, KellyWeingart, Christine L.Laimins, LaimonisWeir, DawnLaitinen, OlliWeiskopf, DanielaLakatos, LorantWeitz, JoshuaLakdawala, SeemaWeitzman, MatthewLam, Tsan YukWendling, Carolin C.Lambert, PaulWennmann, JörgLamp, BenjaminWertheim, JoelLane, MikeWesthaus, SandraLazear, HelenWeynberg, Karen D.Le Henaff, ClaireWhite III, Richard AllenLee, Heung KyuWhite, ElizabethLeitão, AlexandreWhite, KirstenLemay, GuyWhitehurst, ChristopherLemmerman, Niels A.Wiels, JoëlleLennon, JayWilhelm, Steven W.Lenz, OndřejWillemsen, AnoukLePendu, JacquesWilliams, TrevorLeptihn, SebastianWilson, Carolyn A.Leroux, CarolineWilson, Van G.Lesage, PascaleWilusz, JeffLett, Jean-MichelWinckler, ThomasLevin, Henry L.Winston, AlanLevy, LauraWitte, AngelaLeyva-Grado, VictorWobus, ChristianeLi, DapengWodarz, DominikLi, FengWong, Sek-ManLi, Mei-LingWozniakowski, GrzegorzLi, ShitaoWu, Suh-ChinLi, YanWu, YunfengLi, YizeWylie, Stephen J.Licciardello, GraziaXia, NingshaoLieberman, Paul M.Xia, YuchenLin, Chang-ChiXiang, Shi-huaLin, Cheng-WenXiang, YuLin, ShengdaXing, JunjiLindberg, A. MichaelXu, HainengLindenbach, BrettYaegashi, HajimeLindesmith, LisaYamada, ManabuLindsey-Boltz, LauraYang, ZhilongLinial, MaxineYao, Zhi Q.Littlejohn, MargaretYau, ShereeLiu, JiaYeh, Hsin-HungLiu, JinyanYeh, Shiou-HweiLiu, Shan-LuYin, HongLocarnini, StephenYin, LiLodmell, J. StephenYoshida, HiderouLondon, Robert E.You, XiaonaLopalco, LuciaYount, Jacob S.Lopez De Diego, MartaYuan, AdamLópez De Saro, Francisco J.Yutin, NatalyaLopez, CarolinaZajakina, AnnaLopez-Ferber, MiguelZakaryan, HovakimLópez-Huertas, María RosaZakhartchouk, AlexanderLovejoy, ConnieZandi, RoyaLozach, Pierre-YvesZell, RolandLuetgehetmann, MarcZerbini, Francisco M.Luftig, MicahZhang, JianjunLukac, DavidZhang, YijunLundstrom, KennethZhao, JincunLuo, DahaiZhao, WeiLuthra, PriyaZheng, Yong-HuiLuttermann, ChristineZheng, Zhi-MingMa, ZexuZhou, Heshan SamMacDiarmid, RobinZhou, ZhichengMacdonald, AndrewZhu, JiangMacdonald, Noni E.Zhu, LuchangMaeda, NaoyoshiZiebell, HeikoMaeda, YosukeZimmer, GertMager, DixieZitzmann, NicoleMagiorkinis, GkikasZlotnick, AdamMahillon, JacquesZmora, PawelMahomoodally, FawziZúñiga, Sonia

